# *In vivo* and *in vitro* characterization of DdrC, a DNA damage response protein in *Deinococcus radiodurans* bacterium

**DOI:** 10.1371/journal.pone.0177751

**Published:** 2017-05-18

**Authors:** Claire Bouthier de la Tour, Martine Mathieu, Laura Meyer, Pauline Dupaigne, Fanny Passot, Pascale Servant, Suzanne Sommer, Eric Le Cam, Fabrice Confalonieri

**Affiliations:** 1 Institute for Integrative Biology of the Cell (I2BC), CEA, CNRS, Univ. Paris-Sud, Université Paris-Saclay, Gif sur Yvette cedex, France; 2 Signalisations, Noyaux et Innovations en Cancérologie, UMR 8126, CNRS, Univ. Paris-Sud, Université Paris-Saclay, Gustave Roussy, 114 rue E. Vaillant, Villejuif, France; Tulane University Health Sciences Center, UNITED STATES

## Abstract

The bacterium *Deinococcus radiodurans* possesses a set of *Deinococcus*-specific genes highly induced after DNA damage. Among them, *ddrC* (*dr0003*) was recently re-annotated, found to be in the inverse orientation and called *A2G07_00380*. Here, we report the first *in vivo* and *in vitro* characterization of the corrected DdrC protein to better understand its function in irradiated cells. *In vivo*, the Δ*ddrC* null mutant is sensitive to high doses of UV radiation and the *ddrC* deletion significantly increases UV-sensitivity of Δ*uvrA* or Δ*uvsE* mutant strains. We show that the expression of the DdrC protein is induced after γ-irradiation and is under the control of the regulators, DdrO and IrrE. DdrC is rapidly recruited into the nucleoid of the irradiated cells. *In vitro*, we show that DdrC is able to bind single- and double-stranded DNA with a preference for the single-stranded DNA but without sequence or shape specificity and protects DNA from various nuclease attacks. DdrC also condenses DNA and promotes circularization of linear DNA. Finally, we show that the purified protein exhibits a DNA strand annealing activity. Altogether, our results suggest that DdrC is a new DNA binding protein with pleiotropic activities. It might maintain the damaged DNA fragments end to end, thus limiting their dispersion and extensive degradation after exposure to ionizing radiation. DdrC might also be an accessory protein that participates in a single strand annealing pathway whose importance in DNA repair becomes apparent when DNA is heavily damaged.

## Introduction

The *Deinococcus radiodurans* bacterium is known for its exceptional resistance to the lethal effects of ionizing radiation, ultraviolet light and other DNA-damaging agents. Its radioresistance results from a combination of different mechanisms, such as protection of proteins against oxidation, efficient DNA double strand break repair, and a nucleoid structure favoring the maintenance of DNA fragment cohesion after irradiation (for reviews see [1–5]). Transcriptome analysis of cells recovering from exposure to ionizing radiation or desiccation showed complex changes in the expression profile of genes belonging to diverse functional categories [6,7]. Among the 10 most highly up-regulated genes, *ddrA*, *ddrB*, *pprA*, *ddrC* and *ddrD* were specific to *Deinococcacceae*. The DdrA, DdrB, and PprA proteins are DNA binding proteins that have been shown to be involved in radioresistance [7]. They have been extensively characterized *in vivo* and *in vitro*. DdrB promotes a single-strand annealing reaction (SSA), playing an important role for the assembly of small chromosomal fragments produced by high radiation exposure [8–10]. DdrA preferentially binds *in vitro* to 3’ single-stranded DNA ends and was proposed to be part of an end-protection system [11,12]. PprA was shown to protect DNA against degradation by nucleases, to stimulate DNA ligase activity [13] and was recently proposed to be involved in chromosome segregation after completion of DNA repair [14–16]. Single deletion of *ddrD* or *ddrC* gene was described as having no detectable effect on the level of resistance to γ-rays, UV irradiation, or MMC treatment [7,17]. However, when combined with a *ddrB* deletion, the absence of the *ddrC* gene increased significantly the sensitivity to DNA-damaging agents of the single Δ*ddrB* mutant [7,17], suggesting that the DdrC protein might also play a role in DNA damage tolerance.

The *D*. *radiodurans ddrC* gene was first annotated as *dr0003* on the forward strand of *D*. *radiodurans* chromosome 1 [18,19]. More recently, various proteomic and genomic approaches performed on the *D*. *deserti* genome showed that the *ddrC* gene (*Deide_23280*) is encoded by the reverse strand [20]. The corrected *ddrC* gene is now reported under the name *A2G07_003810* after resequencing of the *D*. *radiodurans* R1 genome [21]. In the new orientation, a 17 bp palindromic cis-regulatory element named RDRM (Radiation Desiccation Response Motif), located upstream the coding sequence, was found [20]. A set of about 20 genes up-regulated after irradiation and containing the RDRM sequence in their promoter region were identified and described as part of the RDR regulon [22]. Most of them are involved in the metabolism of DNA. Two proteins, DdrO and IrrE, ensure the regulation of the RDR genes after DNA damage. DdrO is a negative transcriptional regulator that binds the RDRM sequence and represses the expression of the RDR genes [23–25]. IrrE is a metalloprotease [26] that cleaves, and thus inactivates DdrO after irradiation, leading to the transcriptional induction of the genes of the RDR regulon [23,24].

Here, we examined the *in vivo* and *in vitro* properties of the corrected DdrC protein to better understand its function in irradiated cells. We showed that the DdrC protein is induced after γ-irradiation in an IrrE/DdrO dependent manner and is rapidly recruited into the nucleoid of the irradiated cells. *In vitro*, the DdrC protein is able to bind single- and double-stranded DNA (dsDNA) with a preference for the single-stranded DNA (ssDNA). It condenses and protects DNA from nuclease attack, stimulates DNA single-strand annealing and promotes circularization of linearized plasmid DNA.

Taken together, our results show that DdrC is a new DNA binding protein with pleiotropic activities. DdrC might contribute to the repair of radiation induced DNA damage by limiting massive degradation of DNA and by maintaining DNA fragments end to end after exposure to high doses of UV or γ- radiation.

## Material and methods

### Bacterial strains, plasmids, and growth conditions

Bacterial strains and plasmids are listed in S1 Table. *Escherichia coli* strain DH5α was used for cloning of *ddrC* in the pET26b expression vector. Recombinant DdrC-His_6_ protein was expressed in the Rosetta2 (DE3) pLysS strain. All *D*. *radiodurans* strains were derivatives of the wild-type strain R1 ATCC 13939. To construct *D*. *radiodurans* deletion mutants or strains expressing a recombinant tagged protein, the loci of interest were replaced with the appropriate antibiotic resistance cassette or their tagged counterparts, respectively, using the tripartite ligation method [27]. The oligonucleotides used for construction of all these strains are listed in S2 Table. The double mutants were constructed by transformation of a single mutant by the genomic DNA of another single mutant. Genomic DNA of *D*. *radiodurans* was purified and transformation of *D*. *radiodurans* with PCR products or genomic DNA was performed as previously described [8]. The genetic structure and the purity of mutant strains were verified by PCR. Oligonucleotides used for diagnostic PCR and sequencing are available upon request.

*D*. *radiodurans* bacteria were grown at 30°C in TGY2X (1% tryptone, 0.2% dextrose, 0.6% yeast extract) or plated on TGY1X containing 1.5% agar. *E*. *coli* bacteria were grown at 37°C in Luria Broth. Media were supplemented with the appropriate antibiotics used at the following concentrations: hygromycin, 50 μg/ml for *D*. *radiodurans*; chloramphenicol, 35 μg/ml for *E*. *coli* and 3.5 μg/ml for *D*. *radiodurans*; kanamycin, 30 μg/ml for *E*. *coli* and 6 μg/ml for *D*. *radiodurans*; and spectinomycin, 75 μg/ml for *D*. *radiodurans*.

### UV-irradiation of *D*. *radiodurans* bacteria

UV-sensitivity of *D*. *radiodurans* bacteria was tested on plates. For this purpose, cultures of exponentially growing cells at A_650nm_ = 0.3 were serially diluted 1:10 in TGY2X broth and aliquots (10 μl) of each dilution were spotted on TGY1X agar plates. The plates were exposed to different doses of UV-radiation using a UV-C lamp emitting at a calibred dose rate of 3.5 J m^-2^ s^-1^ and incubated at 30°C for 3–5 days.

### γ-irradiation of *D*. *radiodurans* bacteria

A saturated pre-culture was diluted in fresh TGY2X medium and incubated at 30°C to an A_650nm_ = 0.3. Cells were then concentrated to A_650nm_ = 20 and exposed to 5 kGy γ-irradiation on ice (^137^Cs irradiation system GSR-D1, dose rate 18.5 Gy/min, Institut Curie, Orsay). Following irradiation, cells were diluted 100 X in a TGY2X fresh medium and grown at 30°C with shaking. Samples were taken for analysis before irradiation and at the indicated time points after irradiation.

### Western blot analysis of HA-tagged DdrC protein

Non-irradiated or irradiated cultures (5 kGy) of bacteria producing the DdrC-HA protein were diluted in 120 ml TGY2X broth to an A_650nm_ = 0.2 and incubated at 30°C with shaking. Aliquots of 15 ml were taken at different times and centrifugated at 4000 rpm and 4°C. The pellets were resuspended in 150 μl 1X SSC buffer (150 mM NaCl, 15 mM trisodium citrate, pH 7) and cell extracts were prepared as previously described [28]. Proteins from the supernatants were quantified by Bradford assays and 5 μg of crude extracts were resolved on 12% SDS-PAGE gels and transferred onto a PVDF membrane (GE Healthcare). The membrane was incubated overnight at 4°C with a 1:5000 dilution of monoclonal mouse anti-HA antibodies (Eurogentec), and then 1 h at room temperature with a secondary alkaline phosphatase-labeled anti-mouse antibody and revealed by a colorimetric reaction using nitroblue tetrazolium (NBT) and 5-bromo-4-chloro-3-indolyl phosphate (BCIP) as substrates for the alkaline phosphatase (Promega).

### DdrO depletion

GY16917 (Δ*ddrO*/*prepU*_*Ts*_::*ddrO*^+^) were grown at a permissive temperature (30°C) in TGY2X medium supplemented with chloramphenicol and spectinomycin. Cultures at A_650_ = 0.3 were centrifuged and pellets were resuspended in the same volume of fresh culture medium supplemented with chloramphenicol. Then, cells were grown at permissive (30°C) or non-permissive (37°C) temperature for the replication of the *prepU*_*Ts*_::*ddrO+*plasmid. Aliquots of 20 ml were taken for Western blot analyses after 8 h, 16 h and 24 h incubation.

### Localization of GFP-tagged or Cherry-tagged DdrC protein in living cells

The genes coding for GFP and Cherry used in this study, *drGFP* and *drCherry*, are optimized for expression in *D*. *radiodurans* [29] Non-irradiated or irradiated cultures (5 kGy) of cells producing the DdrC-GFP or the DdrC-Cherry proteins were diluted in 10 ml TGY2X to an A_650nm_ = 0.2 and incubated at 30°C with shaking. Aliquots of 500 μl were taken at different times after irradiation and DAPI (2 μg/ml) was added before incubation at room temperature for 5 min. 2 μl of the cell suspension were then immobilized onto 1% agarose coated slides and observed by fluorescence microscopy on a wide-field Leica DM RXA microscope. Images were captured with a CDD camera [5 MHz Micromax 1300Y (Roper Instruments)] equipped with DAPI, GFP and Cherry appropriate filters, and were analyzed with Metamorph and ImageJ softwares.

### Cloning of *ddrC* in an expression vector and protein purification

The *ddrC* coding sequence was PCR-amplified from *D*. *radiodurans* genomic DNA using two primers, DdrC-Nde and DdrC-Xho (S2 Table), and the amplified DNA fragment was cut by *NdeI* and *XhoI* before cloning into pET26b at the same restriction sites. The resulting plasmid (pET26-*ddrC*) expresses a recombinant DdrC protein fused to a 6His-tag at the C-terminal part of the protein. The DNA sequence of the fused *ddrC* gene was verified.

The pET26-*ddrC* plasmid was used to transform *E*. *coli* Rosetta 2 (DE3) pLysS cells. Transformed cells were grown at 37°C in LB medium supplemented with appropriate antibiotics to A_650nm_ = 0.5. After induction of the *ddrC*-6*his* gene expression by 1 mM IPTG during 3 h 30 at 37°C, cells were harvested by centrifugation and the DdrC-6His protein was then purified as previously described by Devigne *et al* [14] for the purification of the GyrA-6His protein.

### Cross-linking of DdrC

DdrC was incubated with 0.1% glutaraldehyde in 10 mM sodium phosphate buffer (pH 7) at 30°C for 30 min in a final volume of 20 μl. After incubation, 5 μl of 5X Laemmli buffer (312.5 mM Tris-HCl pH 6.8, 50% glycerol, 10% SDS, 250 mM DTT, 0.1% bromophenol blue) were added and the samples were analyzed by electrophoresis on 15% SDS-polyacrylamide gel followed by Coomassie blue staining.

### Electrophoretic Mobility Shift Assay (EMSA)

200 ng of supercoiled pBR322 or pBR322 linearized by *PstI* (Thermo-Scientific) (4361 bp), as well as 200 ng of RFI (5386 bp) or single-stranded DNA of phiX174 virion were incubated in 20 μl of buffer A (40 mM Tris-HCl pH 7.8, 5 mM MgCl_2_, 1.5 mM DTT, 50 mM NaCl, 12% glycerol) with increasing concentrations of DdrC (0.86 μM, 1.7 μM, 3.5 μM, 7 μM and 8.6 μM). After 15 min incubation at 4°C, 4 μl of 6X DNA Loading Dye (Fermentas) were added to the mixture before electrophoresis through 1.2% agarose gels. All the non-denaturing electrophoreses were performed in TEP buffer (36 mM Tris-HCl, pH 7.8, 30 mM NaH_2_PO_4_, 1 mM EDTA) at 4.3V/cm for 3 h at 4°C. After staining of the gels with ethidium bromide (1 μg/ml) for 30 min, bands were visualized under UV, using Image Lab (Bio-Rad) software. Binding of DdrC protein to oligonucleotides was performed using a single-stranded 67-mer Cy5 labeled oligonucleotide (oligo-67) or the corresponding double-stranded 67-mer substrate. To produce the ds 67-mer substrate, 1 pmole of oligo-67 and oligo-67 rev were mixed together in a buffer (20 mM Tris-HCl pH 7.5, 50 mM NaCl), heated at 95°C for 2 min, and cooled for 2 h at room temperature. The sequence of the 67-mer was arbitrary selected from the M13 phage genome. The sequences for oligo-67 and oligo-67 rev are: 5’CTGTTTAAGAAATTCACCTCGAAAGCAAGCTGATAAACCGATACAATTAAAGG-CTCCTTTTGGAGCC-3’ and 5’-GGCTCCAAAAGGAGCCTTTAATTGTA-TCGGTTTATCAGCTTGCTTTCGAGGTGAATTTCTTAAACAG-3’, respectively. All reactions were performed in 15 μl of buffer A containing 50 fmoles (3.3 nM) of DNA and increasing concentrations of DdrC (20 nM, 40 nM, 80 nM, 175 nM, 350 nM, 700 nM, 1.4 μM, 2.8 μM). Complexes were separated on 6% (w/v) native polyacrylamide gels (19:1 (w/w) acrylamide/bisacrylamide) in 0.25 X TBE buffer (Tris/Borate/EDTA) containing 10% glycerol. The gels were prerun before loading the reaction mixtures. After migration at 15 V/cm for 135 min at 4°C, bands were visualized by scanning with a Typhoon phosphorimager (Typhoon Trio Imager, GE Healthcare).

### DNA protection assays

The ability of DdrC to protect DNA from digestion by nucleases was assessed on different DNA substrates (supercoiled and linear pBR322, phiX174 single-stranded DNA). Nuclease protection of supercoiled pBR322, linear pBR322 (cut by *EcoRV*) or ss phiX174 virion was tested with 0.1 U DNase I (Promega), 200 U Exonuclease III (NEB) or 1 U Mung Bean Nuclease (Promega), respectively. 200 ng of ds circular DNA, linear plasmid DNA or ss circular DNA were pre-incubated for 15 min at 4°C in the absence or the presence of DdrC (7 μM, 7 μM, and 2 μM, respectively) in 20 μl of buffer A. 2 μl of the respective 10 X nuclease buffer provided by the manufacturer, and nuclease were then added and the samples were incubated at 30°C for 5 min for DNase I, 30 min for Exonuclease III or 15 min for Mung Bean Nuclease. As a control, 200 ng of ds circular pBR322 DNA was incubated simultaneously with 7 μM DdrC and 0.1 U DNase I. After addition of loading buffer, samples were immediately applied onto 1.2% agarose gels. Electrophoreses were performed in TEP 1X buffer at 4.3 V/cm for 3 h at 4°C.

### DAPI (4,6- diamidino-2 phenylindole) fluorescence-based annealing assay

The annealing assay was based on the method described in Kantake *et al* [30]. Briefly, 200 nM of a 67-mer oligonucleotide (oligo-67) were mixed with 0.2 μM DdrC protein, or 0.1 μM T4gp32 (New England Biolabs), or 0.1 μM *E*. *coli* SSB (Sigma), respectively, in 1 ml of a reaction buffer (30 mM Tris-HCl pH 7.5, 5 mM MgCl_2_, 1 mM DTT) containing 0.2 μM DAPI (Thermo Fisher). Reactions were started by addition of the reverse oligonucleotide (oligo-67 rev). The annealing of complementary oligonucleotides was monitored by a Shimadzu RF 6000 spectrofluorometer with excitation and emission wavelengths at 345 and 467 nm using banding of 5 and 10 nm, respectively.

### Effect of DdrC on T4 DNA ligase activity

200 ng of purified pBR322 DNA molecules digested by *PstI* (Thermo-Scientific) were preincubated at 4°C for 15 min in the absence or the presence of increasing concentrations of DdrC protein (0.1 μM, 0.2 μM, 1 μM, 2 μM and 4 μM) prior to addition of T4 DNA ligase (Thermo-Scientific). The reactions were stopped after 15 min at 30°C by addition of a mix of Proteinase K (1 mg/ml) and SDS (0.5%) (10 min at 37°C) and samples were loaded on a 1% agarose gel.

### Transmission electron microscopy (TEM) analysis

PhiX174 single-stranded DNA (ssDNA) (1.4 nM) (New England Biolabs), or supercoiled double-stranded (dsDNA) pBR322 (1.7 nM) (New England Biolabs) was incubated for 30 min at 30°C with DdrC at various concentrations (0.5 μM, 1 μM, or 2 μM) in 10 mM Tris-HCl, pH 7.5, 50 mM NaCl. PhiX174 ssDNA covered with *E*. *coli* Single Strand DNA Binding Protein (SSB, 1 μM) (Sigma) was used as control. To analyze interaction of DdrC with linear DNA fragment, pUC19 plasmid (New England Biolabs) was linearized with *SspI* (New England Biolabs) restriction enzyme producing blunt ends and pBR322 plasmid was linearized with *PstI* (New England Biolabs) producing 3’ overhang cohesive ends. For TEM experiments, all DNA molecules were purified on a MiniQ anion exchange column (GE Healthcare) with a chromatography SMART system. The purified DNA was precipitated and resuspended in 10 mM Tris-HCl, pH 7.5, 1 mM EDTA buffer. DdrC at 0.5 μM or 1 μM was mixed with 2 nM molecule of linear pUC19 DNA fragment or/and pBR322, containing, respectively, blunt and cohesive ends. Nucleoprotein complexes were directly analyzed by TEM. Sample preparations were performed by positive staining, as previously described [31]. Five μl of DNA-protein reaction were deposited onto a 600 mesh copper grid coated with a thin carbon film, previously activated by glow-discharge in the presence of pentylamin (Merck, France). After 1 min, grids were washed with aqueous 2% (w/v) uranyl acetate (Merck, France) and then dried with ashless filter paper (VWR, France). Observations were carried out on a Zeiss 902 transmission electron microscope in filtered annular dark field mode. Electron micrographs were obtained using a Veletta digital camera and the iTEM software (Olympus, Soft Imaging Solutions).

## Results

### Deletion of *ddrC* significantly decreases the UV resistance of *ΔuvsE* mutant

**Fig 1:**
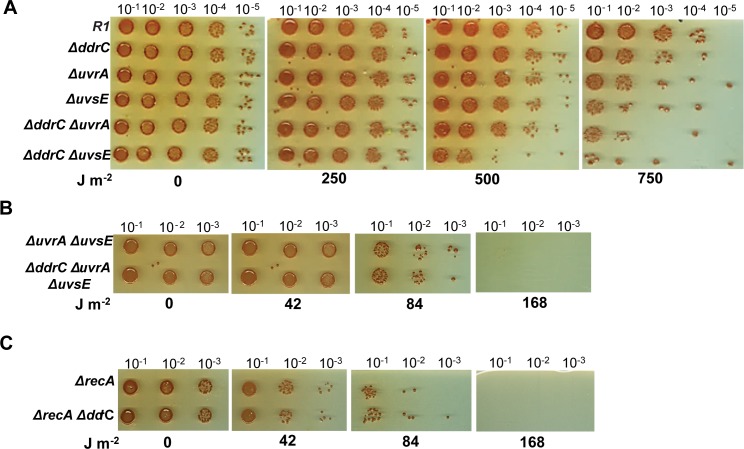
Absence of DdrC increases UV-sensitivity of cells devoid of the UvsE endonuclease. **A** Wild type (R1), Δ*ddrC* (GY 15929), Δ*uvrA* (GY 15971), Δ*uvsE* (GY 15972), Δ*ddrC* Δ*uvrA* (GY 15973), Δ*ddrC* Δ*uvsE* (GY 15974) mutant bacteria grown to an A_650nm_ = 0.3 were serially diluted in TGY2X broth and aliquots (10 μl) of each dilution were spotted on TGY agar plates. Then, the plates were exposed to UV radiation at the indicated UV doses before incubation at 30°C for 3–5 days. **B** Δ*uvrA* Δ*uvsE* (GY 15977) and Δ*ddrC* Δ*uvrA* Δ*uvsE* (GY 15978) and **C** Δ*recA* (GY 15180) and Δ*recA* Δ*ddrC* (GY 15965) mutants were treated as described in Fig 1A. All experiments were performed at least 3 times.

No obvious phenotype was previously described associated with a single *dr0003* locus deletion also resulting in the inactivation of the correctly annotated *ddrC* gene. Here, we show that a single Δ*ddrC* mutant was reproducibly about 10-fold more UV-sensitive than wild-type cells when exposed to high UV-doses, such as 750 J m^-2^ (Fig 1A). Thus, we tested if the DdrC protein is involved in DNA repair of UV-lesions through UvrABC dependent nucleotide excision repair or UVDE repair pathways [32]. For this purpose, a *ddrC* deletion was combined with an *uvrA1* (*dr1771*) or a *uvsE* (*dr1819*) deletion and we measured survival of the resulting strains after exposure to increasing doses of UV-radiation. Strikingly, the absence of the DdrC protein significantly increased UV-sensitivity of a single Δ*uvsE* mutant. Indeed, when exposed to a dose of 500 J m^-2^ UV-radiation, the double mutant Δ*ddrC* Δ*uvsE* is approximately 50-fold more sensitive than the Δ*uvsE* single mutant (Fig 1A). At the same dose, no effect of a *ddrC* deletion was observed on UV-sensitivity of the Δ*uvrA* mutant (Fig 1A). However, when the UV-dose increased to 750 J m^-2^, Δ*ddrC*, Δ*uvrA*, and Δ*uvsE* single mutants were about 10, 100 and 1000 fold more UV-sensitive than the parent R1 strain respectively, and the Δ*ddrC* Δ*uvrA* double mutant was approximately 10 fold more UV-sensitive than the Δ*uvrA* mutant (Fig 1A). The triple mutant Δ*ddrC* Δ*uvrA* Δ*uvsE* exhibited the same UV sensitivity as the double mutant Δ*uvrA* Δ*uvsE* (Fig 1B).

It was previously shown that the *D*. *radiodurans* Δ*recA* mutant was more sensitive to UV radiation than the double mutant Δ*uvrA* Δ*uvsE*, suggesting that the recombinational repair is an essential pathway for replication fork restart in UV-damaged cells [33]. Thus, we analyzed the impact on survival of a *ddrC* deletion in a Δ*recA* mutant, but as shown in Fig 1C, the survival of double mutant Δ*ddrC* Δ*recA* bacteria was identical to those of Δ*recA* mutant bacteria. These results suggest that the DdrC protein might play a role in DNA repair of UV-damaged DNA.

### The expression of the DdrC protein is induced after γ-irradiation in an IrrE- and DdrO-dependent manner

**Fig 2:**
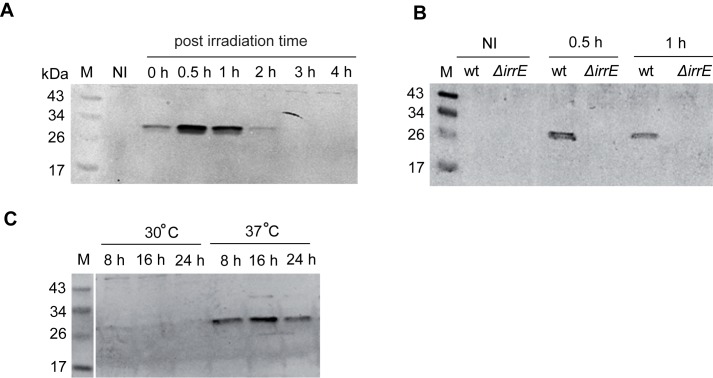
The expression of the DdrC protein was induced after exposure to γ-radiation in an IrrE and DdrO dependent manner. **A and B** GY15921: *ddrC*::*HA* (wt) and GY15967: *ddrC*::*HA* Δ*irrE* (Δ*irrE*) bacteria exposed or not to 5 kGy γ-radiation were diluted to an A_650nm_ = 0.2 and incubated at 30°C for the indicated periods (hours). Cell extracts were subjected to SDS-PAGE and analyzed by Western blot with anti-HA antibodies. 5 μg of proteins were loaded on each well. Lane NI: non-irradiated cells. Lane 0 h: non-incubated irradiated cells. **C** GY16917: a [*ddrC*::*HA* Δ*ddrO* (p*repU*_*Ts*_ *ddrO*^+^)] culture grown at 30°C in TGY2X broth supplemented with spectinomycin (A_650nm_ = 0.3) was divided into two identical vials and incubated at 30°C or at 37°C, respectively for the indicated periods (hours). Cell extracts were subjected to SDS-PAGE and analyzed by Western blotting with anti-HA antibodies. Ten μg of proteins were loaded on each well.

The newly annotated *A2G07_003810* gene [21], located on the opposite strand of *dr0003* [19], encodes a DdrC protein of 231 amino acids (deduced MW: 25.148 Da, IP: 10.8) (S1 Fig) that exhibits 70% identity with the *deide_23280 D*. *deserti* homolog. An RDRM sequence, found in the promoter regions of genes induced upon radiation/desiccation [22], was located 19 nt upstream the putative start codon of *A2G07_003810* [20]. Thus, we investigated the expression of a C-terminal HA-tagged DdrC protein after exposure of *D*. *radiodurans* cells to a dose of 5 kGy γ-irradiation and tested if it was under the control of the IrrE and DdrO regulatory proteins (Fig 2).

Western blot analyses (Fig 2A) showed that the DdrC-HA protein was expressed at an undetectable level under normal growth conditions, but accumulated early within the cells in response to radiation-induced DNA damage, with a maximum expression at 0.5–1 h after irradiation. In contrast, no signal was detected in the Δ*irrE* mutant (Fig 2B), suggesting that, *in vivo*, IrrE is directly or indirectly involved in the induction of the DdrC expression in response to irradiation. We then tested whether the DdrO regulator controlled the expression of the DdrC protein. As shown in Fig 2C, depletion of the DdrO protein in cells expressing *ddrO* from a prepU_Ts_ plasmid and grown at 37°C, a non-permissive temperature for the replication of the plasmid, resulted in an increase of the cellular level of the DdrC-HA protein. Thus, the degradation of the DdrO regulator by the IrrE protease that occurred after irradiation [24] leads to the induction of the DdrC protein expression.

### The DdrC protein is recruited to the nucleoid after exposure to γ-irradiation

To determine the cellular localization of the DdrC protein, we replace the *ddrC* gene by its *drGFP*- or *drCherry*-tagged counterpart. We first verified the functionality of the tagged proteins in cells devoid of the UvsE protein. As shown in S2 Fig, *ddrC*::*drGFP* Δ*uvsE* and *ddrC*::*drCherry* Δ*uvsE* bacteria were more resistant to UV than Δ*ddrC* Δ*uvsE* double mutant bacteria, suggesting that the tagged DdrC-GFP or DdrC-Cherry proteins remained functional. Then, we determined the cellular localization of the tagged DdrC proteins by fluorescence microscopy at different times after exposure to a dose of 5 kGy γ-irradiation.

**Fig 3:**
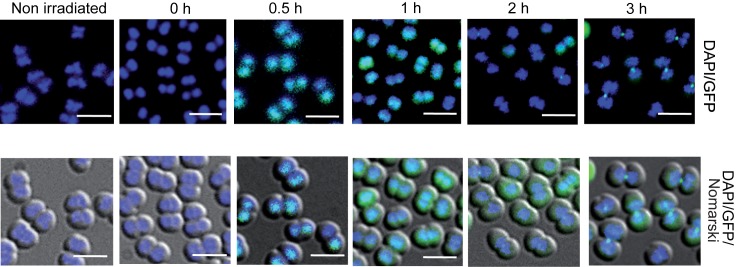
Cellular localization of DdrC-GFP after γ-irradiation of *D*. *radiodurans* cells. Bacteria expressing a DdrC-GFP fusion protein (GY15931) recovering from γ-irradiation (5 kGy) were visualized by fluorescence microscopy at the indicated times of post-irradiation incubation. DNA was stained with DAPI. Overlays of GFP (green) and DAPI (blue) images as well as overlays of Nomarski DIC (grey), GFP and DAPI are shown.

Interestingly, the GFP-tagged DdrC protein was transiently associated with the nucleoid in more than 90% of the observed cells after a 0.5 h (199/200) and 1 h (182/201) postirradiation incubation (Fig 3). The proportion of fluorescent cells dropped to 42% (88/208) at 2 h. The localization of the DdrC protein changed with time with foci at the septum of dividing cells observed in 24% and 68% of cells after 2 h and 3 h post irradiation incubation, respectively. We observed the same patterns of fluorescence in cells expressing a C-terminal Cherry-tagged DdrC protein (S3 Fig). These data suggest that DdrC protein might interact with damaged DNA after irradiation.

### DdrC exists both as a monomer and a dimer in solution

**Fig 4:**
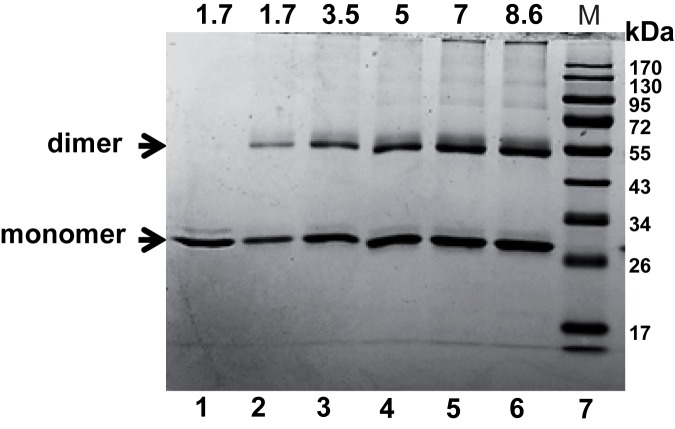
Dimerization of DdrC in solution. Lane 1: Purified recombinant DdrC-His_6_ protein. Lanes 2–6: Increasing concentrations of recombinant DdrC protein (μM) crosslinked with glutaraldehyde. Lane 7: Molecular weight markers (kDa).

In order to investigate the biochemical properties of the DdrC protein, the correct *ddrC* gene was cloned in the pET26b *E*. *coli* expression vector with a C-terminal 6X His-tag. As seen in SDS-PAGE analysis, the purified recombinant protein was found to have an approximate MW of 26 kDa (Fig 4) correlating with the deduced amino acid sequence (26.2 kDa) and we verified that the recombinant protein was functional *in vivo* (S2 Fig). Using glutaraldehyde as a crosslinking agent, an intense band migrating at about 50 kDa was observed on the SDS PAGE gel, likely corresponding to the dimeric form of DdrC. Thus, DdrC was mainly present both in monomeric and dimeric forms in solution, even if several faint bands attributed to multimeric forms of DdrC were visible after glutaraldehyde treatment when the DdrC amount was > 2.7 μg.

### DdrC binds to DNA *in vitro* with a preference for ssDNA

**Fig 5:**
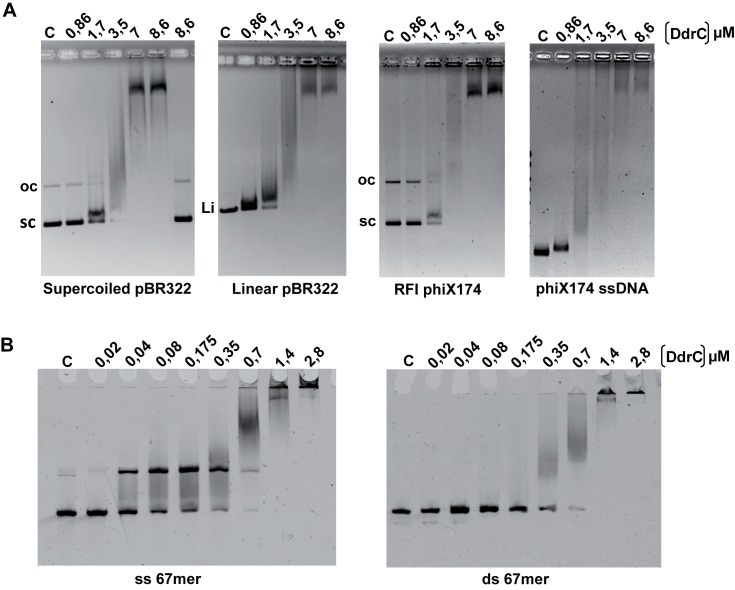
DdrC binds to ssDNA and dsDNA with a preference for ssDNA. **A** Binding of recombinant DdrC to plasmid or viral DNA analyzed by EMSA. 200 ng of supercoiled or linear pBR322 DNA as well as 200 ng of RFI or single-stranded DNA of phiX174 virion (31 μM nucleotides of each DNA) were incubated with increasing concentrations of DdrC as indicated in the figure. DNA-protein complexes were separated in 1.2% agarose gels. Products loaded in the right lane of the left panel were treated with SDS and proteinase K. sc: supercoiled dsDNA, oc: open circle dsDNA, Li: linear dsDNA. **B** Binding of DdrC to oligonucleotides. Increasing concentrations of DdrC were incubated with 3.3 nM of a single-stranded (ss) 67-mer fluorescent oligonucleotide (left panel) or 3.3 nM of the corresponding ds oligonucleotide (right panel). The products of the reactions were separated in 6% native polyacrylamide gels. Lanes C: DNA control without DdrC.

Since DdrC is recruited onto the nucleoid after irradiation, we investigated by EMSA assay the ability of His_6_-tagged DdrC proteins to bind DNA *in vitro*. DdrC induced a total DNA shift when 3.5 μM of protein was incubated with negatively supercoiled pBR322 plasmid DNA (Fig 5A). At higher concentrations, DdrC binding resulted in large DNA-protein complexes. The treatment of these nucleoprotein complexes with proteinase K and SDS released intact DNA, indicating that the DNA had not undergone any covalent modification (Fig 5A, left panel, lane 6).

The DNA shift promoted by DdrC was also observed with linear pBR322 DNA and with supercoiled phiX174 dsDNA (RFI) (Fig 5A) showing no preference for the different topological forms of DNA. However, when the single-stranded DNA form of phiX174 (phiX174 ssDNA) was used as a substrate, a complete shift was visible at 1.7 μM of the DdrC protein, while at this protein concentration a band corresponding to the unbound dsDNA substrate (RFI form) was still visible.

This greater affinity for ssDNA than for dsDNA was further analyzed by comparing the binding of DdrC to ss and ds 67-mer oligonucleotides (Fig 5B). A discrete band corresponding to ssDNA-DdrC complex appeared at 40 nM DdrC, while no band shift was observed with dsDNA at the same DdrC concentration. Finally, we showed that DdrC also binds with the same efficiency to 81-mer oligonucleotides containing a G+C percentage of 37% or 65.4% (S4 Fig). Taken together, these results suggest that DdrC protein exhibits a preference for the ssDNA substrate without sequence specificity.

### DdrC protects DNA from degradation by nucleases

**Fig 6:**
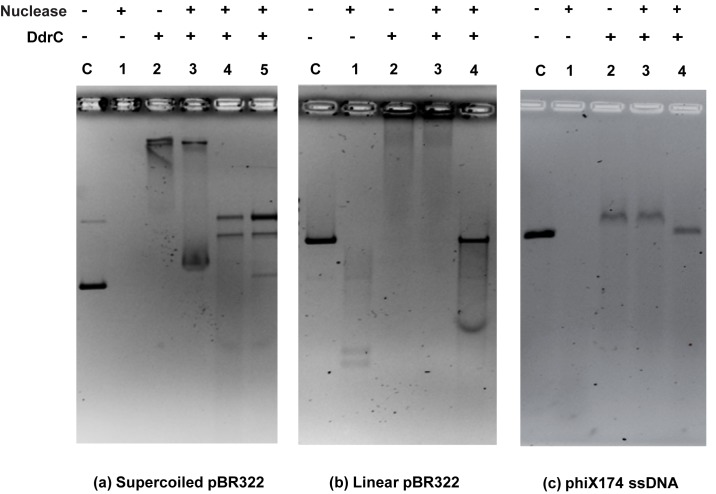
DdrC protects DNA against degradation by nucleases. Protection of supercoiled pBR322 plasmid (3.5 nM) from DNase I activity (0.1 U) (panel a), linear pBR322 (3.5 nM) from Exonuclease III activity (200 U) (panel b) and phiX174 ssDNA (5.9 nM) from Mung Bean Nuclease activity (1 U) (panel c) by 7 μM, 7 μM, and 2 μM DdrC, respectively. Lanes C: DNA controls without protein. Lanes 1: DNA incubation with nuclease alone. Lanes 2: DNA incubation with DdrC alone. Lanes 3: DNA pre-incubated with DdrC 15 min at 4°C before addition of nuclease. Lanes 4: Reaction products corresponding to lane 3 were further treated with Proteinase K/SDS. Panel a, lane 5: DdrC and DNase I were simultaneously incubated with supercoiled DNA before treatment with Proteinase K/SDS.

Then, we investigated the DdrC ability to protect DNA from nucleases. DdrC was incubated with different DNA substrates prior to the addition of endo- or exo-nucleases (Fig 6). Whereas massive DNA degradation was observed in the absence of DdrC (Fig 6, lanes 1 of panels a, b, c), the presence of DdrC substantially protected supercoiled pBR322, linear pBR322 and closed circular ssDNA from degradation by DNase I endonuclease, exonuclease III and Mung Bean endonuclease, respectively (Fig 6, lanes 3 of panels a, b and c). A further treatment with proteinase K and SDS released intact DNA substrate molecules (Fig 6, lanes 4 of panels a, b and c). Interestingly, when DdrC and the DNase I are simultaneously added to the supercoiled pBR322 plasmid, the products were similar to those observed when DNA was preincubated with the DdrC protein (Fig 6, lane 5 of panel a) suggesting that DdrC binds rapidly to DNA and impedes access of DNase I to DNA, thus preserving DNA from degradation.

### DdrC stimulates annealing of complementary DNA strands

**Fig 7:**
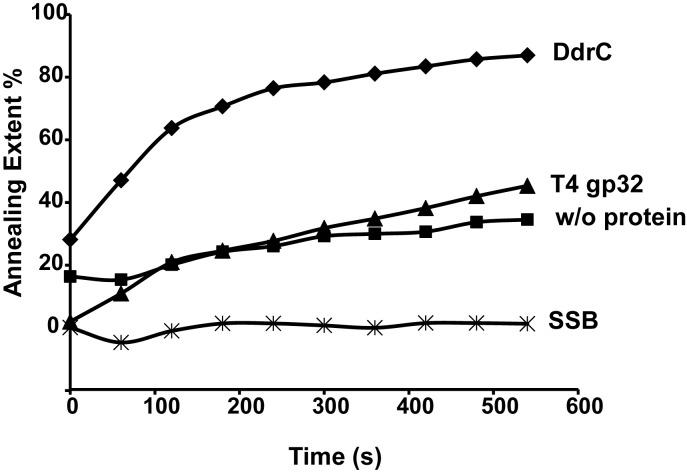
DdrC stimulates DNA annealing. Kinetics of two complementary 67-mer oligonucleotides annealing in the absence (w/o protein) or the presence of DdrC, T4 gp32 or SSB using a DAPI fluorescence-based method. The 67-mer oligonucleotide (200 nM) was mixed in 1 ml of reaction buffer with 0.2 μM DdrC protein, or 0.1 μM T4 gp32, or 0.1 μM SSB from *E*. *coli* prior to addition of the reverse oligonucleotide. The extent of DNA annealing is defined as follows: (observed fluorescence—67-mer ssDNA fluorescence) x 100 / 67-mer ds DNA fluorescence.

As DdrC preferentially binds ssDNA (Fig 5), we also tested if DdrC promotes DNA strand annealing. For this purpose, we examined the annealing of two complementary 67-mer oligonucleotides in presence or absence of DdrC using a DAPI fluorescence-based method [30]. In the absence of any protein, only a few spontaneous annealings occurred over time, resulting in a very slow increase of dsDNA-specific DAPI fluorescence (Fig 7, without protein). As a control, we also verified that *E*. *coli* SSB protein did not stimulate the formation of DNA duplex (Fig 7, SSB). In contrast, when DdrC protein was mixed with the 67-mer oligonucleotide, addition of the complementary oligonucleotide resulted in a rapid increase in DAPI fluorescence (Fig 7, DdrC), as observed with the T4gp32 protein known to stimulate efficiently DNA annealing [30] (Fig 7, T4gp32). Thus, we conclude that the DdrC protein exhibits a single-strand annealing activity.

### DdrC condenses DNA *in vitro*

**Fig 8:**
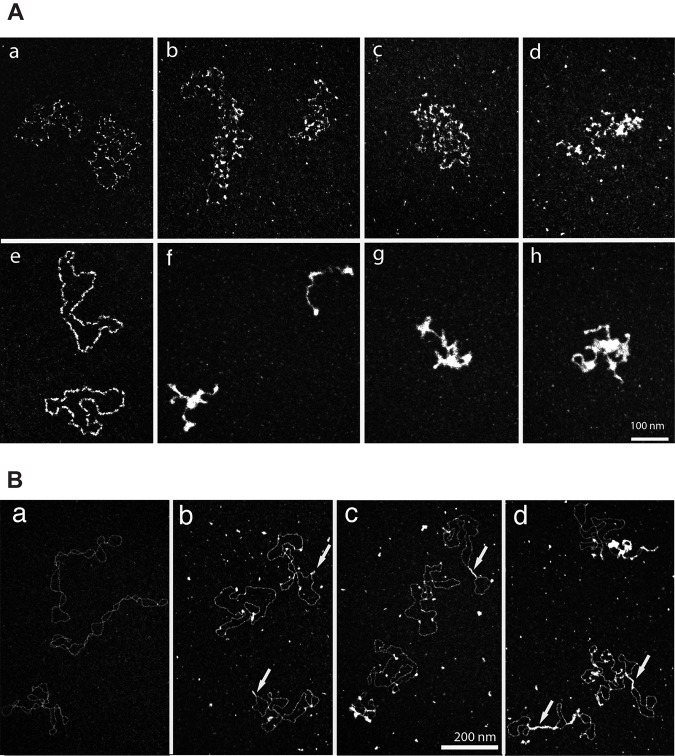
Visualization of DdrC-DNA complexes by transmission electron microscopy. **A** PhiX174 ssDNA (1.4 nM, 7.5 μM nucleotides) was incubated with 1 μM (panels b-d) or 2 μM (panels f-h) of DdrC. Panel a: phiX174 ssDNA control without DdrC. Panel e: Interaction of *E*. *coli* SSB protein (1 μM) with ssDNA. Magnification = 85,000. **B** Supercoiled pBR322 DNA (1.7 nM, 7.5 μM base pairs) incubated with 1 μM (panel b and c) or 2 μM (panel d) of DdrC. Panel a: pBR322 DNA control without protein. Magnification = 85,000. Some“bridge” structures, forming loops or kinks, are indicated by arrows.

To further investigate DdrC-DNA interactions, transmission electronic microscopy (TEM) was performed with various DNA substrates (circular phiX174 ssDNA, supercoiled pBR322 dsDNA) (Fig 8). As shown in Fig 8A (panel e), the *E*. *coli* SSB protein, at a 1:7.5 protein /nucleotide ratio, coats and stretches ssDNA in the “*E*. *coli* SSB_35_” binding mode, removing the DNA secondary structures as previously described [34]. At the same ratio, DrdC interacted with ssDNA and slightly compacted it (Fig 8A, panels b, c, d). At a higher protein concentration (1:3.75 ratio), highly compacted structures were observed (Fig 8A, panels f, g, h) suggesting protein-protein interactions resulting in a tangled network of DdrC-DNA complexes. When DdrC was incubated with negatively supercoiled pBR322 DNA, “bridge” structures were observable, leading to the formation of loops or kink-like structures (Fig 8B, panels b to d). Relaxed forms were observed at a 1:7.5 protein / base pair ratio (Fig 8B, panels b and c) while at higher DdrC concentration (1:3.75 protein / nucleotide ratio), condensed forms were visible (Fig 8B, panel d).

### DdrC protein promotes circularization of linear plasmid DNA

**Fig 9:**
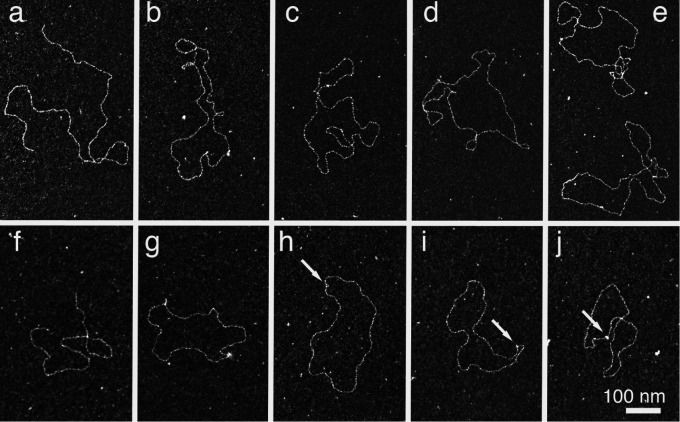
Circularization of pBR322 (cohesive ends) and pUC19 (blunt ends) plasmids mediated by DdrC visualized by electron microscopy. Panel a: Control pBR322 DNA linearized by PstI. Panels b-e: pBR322 circularization mediated by DdrC. Panel f. Control pUC19 DNA linearized by Ssp11. Panels g-j: pUC19 circularization mediated by DdrC. 1 μM DdrC was mixed with 2 nM molecules of linear pBR322 or pUC19 plasmid, containing cohesive or blunt ends, respectively. The shapes are similar at 0.5 μM, 1 μM or 2 μM of DdrC. Magnification = 85,000. Some loci of plasmid circularization are indicated by arrows.

TEM was also performed with linearized pBR322 and pUC19 plasmids with cohesive and blunt ends, respectively (Fig 9). Strikingly, when DdrC was incubated with linear plasmids containing cohesive or blunt ends, circularized DNA were observed, showing that the DdrC protein was able to join the both DNA ends (Fig 9 panels b-e) for pBR322 and panels g-j for pUC19). Various events were also detected such as loops or kink-like structures related to the ability of DdrC to bridge DNA (Fig 9) as we observed in Fig 8. However, we cannot assert that the positions of the kinks observed in Fig 9 coincide with the loci of plasmid circularization. The percentage of plasmid circularization observed for linear pBR322 containing cohesive ends was 28%, 52% and 84% at 0.5 μM, 1 μM and 2 μM DdrC concentrations, respectively. When linear pUC19 plasmid containing blunt ends was incubated with DdrC at the same concentrations, similar percentages of circularized DNA were found (24%, 47%, and 78%, respectively). Each observation was carried out with a set of 300 molecules.

Since DdrC was able to circularize linear DNA molecules (Fig 8), we tested whether DdrC could stimulate DNA ligation activity as does *D*. *radiodurans* PprA protein [13]. We found that DdrC protein did not stimulate ligation of pBR322 digested with *PstI* when incubated with different concentrations of T4 DNA ligase (S5 Fig). T4 DNA ligase activity appeared to be inhibited when DdrC was added at concentrations > 1 μM (S5 Fig) suggesting that DdrC might prevent access of the ligase to DNA.

## Discussion

The *D*. *radiodurans* bacterium possesses an exceptional ability to tolerate massive DNA damage generated by exposure to high doses of UV or γ-radiation. It was previously shown that some genes specific to the *Deinococcaceae*, including *ddrA*, *pprA* and *ddrB*, are highly induced after irradiation and involved in radioresistance [7]. In contrast, no obvious phenotype was associated, to date, with the single deletion of the *ddrC* (*dr0003*) gene that yet was shown to be induced 12-fold when cells were exposed to 3 kGy γ-radiation [7]. More recently, it was proposed that the orientation of *ddrC* was incorrect [20] and the *ddrC* gene was reannotated [21]. The newly annotated *ddrC* gene, called *A2G07_003810*, encodes a small basic protein with an apparent MW of approximately 25 kDa. An RDRM sequence was found 19 nt upstream the putative start codon of *A2G07_003810* (S1 Fig). DdrC gene has only homologs in bacteria belonging to *Deinococcaceae* and the encoded protein does not contain any predictable motif that might suggest a function for this protein. Here, we examined the regulation of the newly annotated DdrC protein, its localization in cells and we investigated its biochemical properties.

To better understand the *in vivo* functions of the DdrC protein, we first deleted the *ddrC* gene and showed that cells devoid of the DdrC protein were 10 times more sensitive to UV-radiation than wild-type cells when exposed to UV-doses higher than 750 J m^-2^. Moreover, the *ddrC* deletion significantly increased UV-sensitivity of Δ*uvrA* or Δ*uvsE* mutant cells, but not UV sensitivity of the Δ*uvrA* Δ*uvsE* double mutant, or Δ*recA* mutant cells. We propose several hypotheses to explain these phenotypes: the DdrC protein might be involved in both nucleotide excision repair (NER) and UVDE pathways or in recombinational repair of UV-lesions that takes place when the replication fork encounters residual unrepaired UV-lesions. DdrC might also participate in DNA repair of numerous DNA double strand breaks generated by exposure of cells to high doses of UV. Finally, DdrC might have an indirect effect on survival by protecting DNA against degradation.

We showed that the cellular concentration of DdrC is increased after irradiation, in a manner strongly dependent on the presence of the IrrE metalloprotease [26]. Moreover, the depletion of the DdrO protein results in an increase of the cellular concentration of the DdrC protein, suggesting that the *ddrC* gene belongs to the RDR regulon. Interestingly, it was also proposed that the *ddrC* gene could be a target of the DrRRA protein, another important response regulator involved in radioresistance [35]. Therefore, the *ddrC* gene expression might be directly or indirectly under the control of several regulators, allowing a fine tuned expression of this gene in the cell. Recent analysis of RNA sequencing and proteomics performed in *D*. *deserti* [36] revealed, that its *ddrC* mRNA transcript was leaderless. To date, we do not have any evidence that the *A2G07_003810* mRNA is also leaderless.

Studies of cellular dynamics of DdrC protein showed that it was rapidly recruited into the nucleoid after exposure to γ-rays, suggesting that it interacts with DNA. We confirmed this by showing that, *in vitro*, the DdrC protein is able to interact with both ssDNA and dsDNA, with a binding preference for ssDNA and without DNA sequence specificity. Moreover, DdrC protected ssDNA and dsDNA against degradation by endo- or exonucleases. Furthermore, a pre-incubation of DdrC protein with supercoiled DNA was not a prerequisite to prevent DNA degradation from endonuclease, highlighting a rapid and efficient formation of nucleoprotein complexes. Other proteins, whose expression increased after irradiation, were also able to protect DNA from nuclease attack, as shown for DdrA which protects 3’ single-stranded DNA ends against digestion by exonuclease I [11] and for PprA which protects linear dsDNA from degradation by exonuclease III [13]. It was previously shown that transient DNA degradation was observed rapidly after exposure of the cells to ionizing radiation [37] but this degradation has to be controlled to ensure DNA double strand break repair and cell survival. Thus, DdrC might prevent, along with DdrA and PprA, extensive DNA degradation of severely damaged DNA. Mattimore and Battista [38] suggested that *D*. *radiodurans* resistance to ionizing radiation is a consequence of its adaptation to dehydration. In the desiccated state, nucleases may still function whereas DNA repair processes requiring ATP may be inefficient. Thus, proteins able to protect DNA from nuclease attack such as DdrC, PprA, and DdrA [11,13], specific from *Deinococcaceae*, and highly induced after exposure to dessication [7], may play an important role in the adaptation to dehydration.

Interestingly, when the DdrC protein was incubated with linearized plasmid DNA containing cohesive or blunt ends, re-circularized DNA was observed by transmission electron microscopy, suggesting that DdrC brings closer together the dsDNA ends by protein-protein interactions. To our knowledge, the spectacular efficiency of DdrC to circularize linear DNA has never been reported for any other *Deinococcus* protein, including PprA [39]. However, whereas the PprA protein was able to stimulate DNA ligase activity [13], we did not observe any evidence of the stimulation of DNA ligation by the DdrC protein.

Another feature shared by the PprA and the DdrC proteins is their rapid recruitment into the nucleoid after exposure to γ-radiation and their re-localization at the septum after completion of DNA repair [15]. However, even if PprA and DdrC share some properties, their absence does not have the same consequences on cell viability. In particular, cells devoid of the PprA protein are highly sensitive to ionizing radiation and exhibit a characteristic cell division abnormality after irradiation whereas cells devoid of the DdrC protein are as resistant to γ-rays as wild type cells [7]. Moreover, sensitivity to ionizing radiation of Δ*pprA* bacteria was slightly decreased when a *ddrC* deletion was combined with the *pprA* deletion [7]. Thus, the role of the apparent functional redundancies of the PprA and DdrC proteins in *Deinococcus* cells remains questionable.

Several properties of the DdrC protein are also reminiscent of those of the DdrB protein. Both proteins are rapidly recruited to the nucleoid after irradiation, and, *in vitro*, they bind with high affinity single-stranded DNA and promote annealing of complementary single-stranded DNA [9,10,40]. Moreover, the Δ*ddrC* Δ*ddrB* double mutant was previously shown to be more sensitive to high doses of UV (1000 J m^-2^) or γ-rays (10 kGy) than the single Δ*ddrB* mutant [7,17] suggesting that the DdrC protein may partially compensate *in vivo* the absence of DdrB. It has been proposed that the DdrB protein facilitates the assembly of a myriad of small fragments by a single strand annealing (SSA) process [8].

However, DdrB and DdrC do not share all their properties. The DdrB protein is an SSB-like protein that coats ssDNA [40]. Here, we show by TEM that the DdrC protein does not coat ssDNA but condenses it, leading to highly compacted structures when DdrC is present at high concentrations. The DdrC protein also binds dsDNA and we observed, by TEM, the formation of bridge and kink-like structures when the DdrC protein was incubated with supercoiled and linear DNA molecules, respectively. This suggests that DdrC may promote the juxtaposition of independently bound DNA molecules, likely via protein-protein interactions. The ability of DdrC to recognize single-strand regions within supercoiled DNA may also contribute to the formation of the bridged structures.

*In vitro* characterization of DdrC indicates that the DdrC protein is likely involved in several pathways of DNA metabolism. Redundancies of protein activities in DNA damage responses make difficult to determine an obvious role of DdrC in the ability of *D*. *radiodurans* to tolerate high levels of DNA damage. It might rapidly condense DNA after irradiation, maintain DNA fragments end to end, and thus limit dispersion of damaged DNA fragments and their extensive degradation. Through its ability to bind ssDNA and anneal complementary DNA strands, DdrC might also be an accessory protein that favors single strand annealing and homology search by the RecA protein. Further structural studies on DdrC protein and the search of DdrC partners will be necessary to unravel the function of DdrC *in vivo* and to provide clues about how this protein recognizes ssDNA and dsDNA, drives annealing of DNA complementary strands, juxtaposes independently bound molecules and favors circularization of linear DNA molecules.

## Supporting information

S1 Fig *D*. *radiodurans A2G07_003810* locus sequence. (PDF)

S2 Fig Cells expressing His6-tagged, GFP-tagged and Cherry-tagged DdrC proteins are functional. (PDF)

S3 Fig Cellular localization of DdrC-Cherry after γ-irradiation of *D*. *radiodurans* cells. (PDF)

S4 Fig DdrC binds to ssDNA without preference of DNA sequence. (PDF)

S5 Fig DdrC protein does not stimulate DNA ligation of cohesive ends by T4 DNA ligase. (PDF)

S1 Table Bacterial strains and plasmids. (PDF)

S2 Table Oligonucleotides used for strain constructions. (PDF)
