# Supplementary material for: In vivo and in vitro characterization of DdrC, a DNA damage response protein in Deinococcus radiodurans bacterium
Source: PLoS One. 2017 May 18;12(5):e0177751. doi: 10.1371/journal.pone.0177751 (PMC5436757; doi:10.1371/journal.pone.0177751)
Supplement: S1 Fig — (PDF) [file pone.0177751.s001.pdf]

A

```
gccttcagcg aactttaaga taattttctc cgtcctgcac actgaaaaat agccctgctt

ccgtgctgat ttgttatgtc aaaaacataa tctgtgctag aatatctgt atgaagaacgc
                   RDRM                                m  k  n

tccgctgacc ctcaatttcg gctccgtgcg gctgcctgtc agcgcggacg gtttgcttca
a  p  l  t   l  n  f   g  s  v   r  l  p  v   s  a  d   g  l  l

cgccccccacc gcccagcagc agctcgggct cacccaaagc tgggaagcgg cgctggtcga
h  a  p  t   a  q  q   q  l  g   l  t  q  s   w  e  a   a  l  v

acacggcctg cccgagacgt accgcgactt cggcgccgga cccgaggccg ccgtgagcgt
e  h  g  l   p  e  t   y  r  d   f  g  a  g   p  e  a   a  v  s

gcccgatttc gtggcgcttg ccttcgccct cgacaccccc gaagcccggc gctggcagaa
v  p  d  f   v  a  l   a  f  a   l  d  t  p   e  a  r   r  w  q

gcgggcgcgg gagctgctcg cccgcgccat gcagggcgac gtgcgcgtgg ctgcccagat
k  r  a  r   e  l  l   a  r  a   m  q  g  d   v  r  v   a  a  q

tgccgagcgc aaccccgagc ctgacgcccg gcgctggctc gctgcccgac tggaaagcac
i  a  e  r   n  p  e   p  d  a   r  r  w  l   a  a  r   l  e  s

tggcgcccgg cgcgaactgc tcgccaccgt cgcccggcac ggtggcgagg gccgggtcta
t  g  a  r   r  e  l   l  a  t   v  a  r  h   g  g  e   g  r  v

cggtcagctc ggcagcatca gcaaccgcac cgtccttggc aaggacagcg cgtcggtacg
y  g  q  l   g  s  i   s  n  r   t  v  l  g   k  d  s   a  s  v

tcaggaacgc ggcgtcaagg ccacccgcga cggcctgacg agcgccgaac tgctgcggct
r  q  e  r   g  v  k   a  t  r   d  g  l  t   s  a  e   l  l  r

ggcctacatc gacaccgtga ccgcccgcgc cattcaggaa agcgaagcgc ggggcaacgc
l  a  y  i   d  t  v   t  a  r   a  i  q  e   s  e  a   r  g  n

agccatcctc accctccacg aacaggtagc ccgcagcgag cgccagagct gggaacgggc
a  a  i  l   t  l  h   e  q  v   a  r  s  e   r  q  s   w  e  r

gggccaggtg cagcgcgtag gctgagacaa gcgccataaa gaagctgccc ctatcttcca
a  g  q  v   q  r  v   g  STOP
```

B

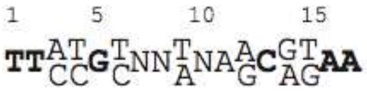

RDRM consensus

S1 Fig. ***D. radiodurans A2G07_003810*** **locus sequence**

A Nucleotide sequence of the *A2G07_003810* locus and deduced amino acid sequence of DdrC protein. The putative start codon is in bold and the RDRM motif is underlined. **B** Consensus sequence of RDRM sites (from [23]).
