# Supplementary material for: In vivo and in vitro characterization of DdrC, a DNA damage response protein in Deinococcus radiodurans bacterium
Source: PLoS One. 2017 May 18;12(5):e0177751. doi: 10.1371/journal.pone.0177751 (PMC5436757; doi:10.1371/journal.pone.0177751)
Supplement: S2 Fig — (PDF) [file pone.0177751.s002.pdf]

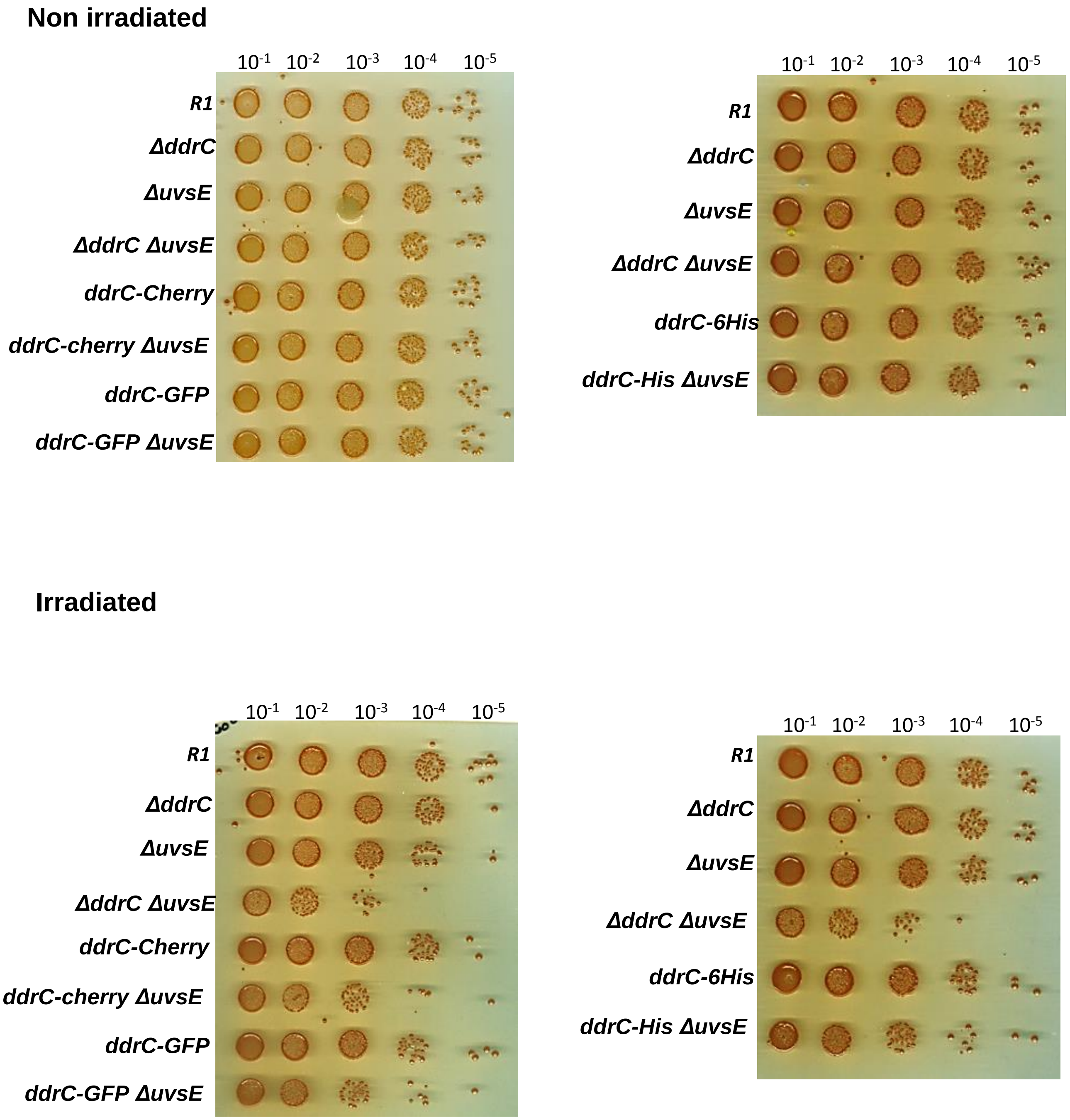

S2 Fig. Cells expressing His6-tagged, GFP-tagged and Cherry-tagged DdrC proteins are functional. Wild type (R1), Δ*ddrC* (GY15929), Δ*uvsE* (GY15972), Δ*ddrC* Δ*uvsE* (GY15974), *ddrC*::*Cherry* (GY15928), *ddrC*::*Cherry* Δ*uvsE* (GY16905), *ddrC*::*GFP* (GY15931), *ddrC*::*GFP* Δ*uvsE* (GY16906), *ddrC*::*6His* (GY16901), *ddrC*::*6His* Δ*uvsE* (GY16902) bacteria grown to an A_{650nm}= 0.3 were serially diluted in TGY2X broth and aliquots (10 µl) of each dilution were spotted on TGY agar plates. Then, the plates were exposed to 500 J m^{-2} UV radiation before incubation at 30°C for 3-5 days.
