# Supplementary material for: In vivo and in vitro characterization of DdrC, a DNA damage response protein in Deinococcus radiodurans bacterium
Source: PLoS One. 2017 May 18;12(5):e0177751. doi: 10.1371/journal.pone.0177751 (PMC5436757; doi:10.1371/journal.pone.0177751)
Supplement: S3 Fig — (PDF) [file pone.0177751.s003.pdf]

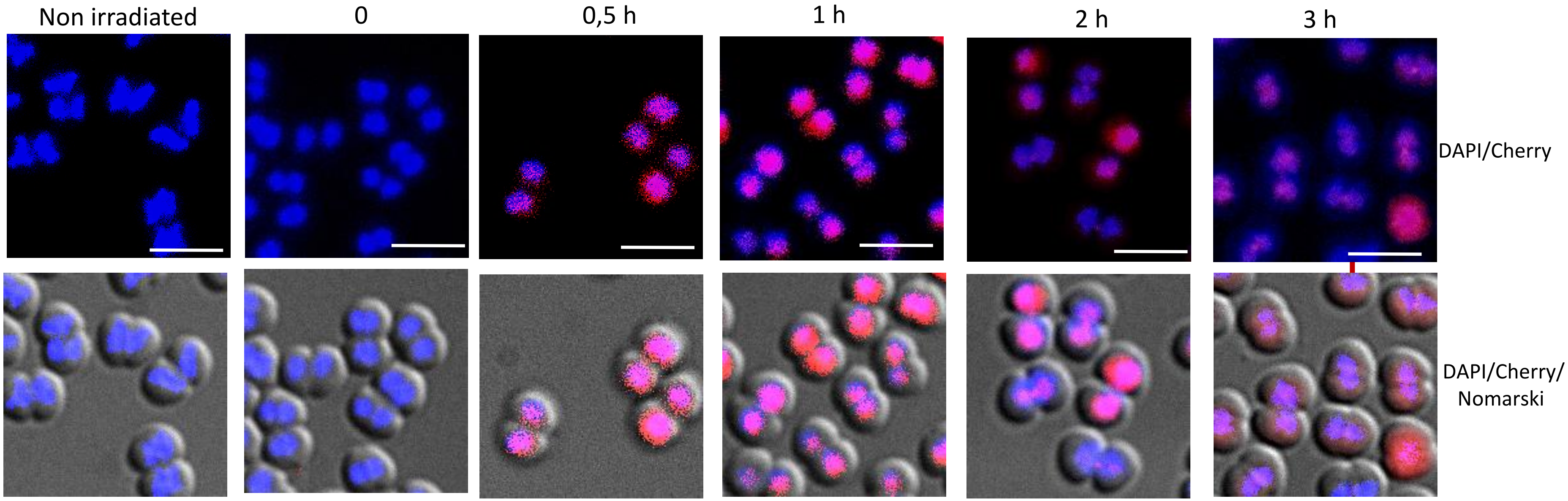

S3 Fig. Cellular localization of DdrC-Cherry after γ-irradiation of *D. radiodurans* cells.

Samples of irradiated cells (5 kGy) expressing the Cherry-tagged DdrC (GY15928) were taken at the indicated post-irradiation times and visualized by fluorescence microscopy (pink). DNA was stained with DAPI (blue). Overlays of Cherry and DAPI images, as well as overlays of Nomarski DIC, Cherry and DAPI are shown.
