# Supplementary material for: In vivo and in vitro characterization of DdrC, a DNA damage response protein in Deinococcus radiodurans bacterium
Source: PLoS One. 2017 May 18;12(5):e0177751. doi: 10.1371/journal.pone.0177751 (PMC5436757; doi:10.1371/journal.pone.0177751)
Supplement: S4 Fig — (PDF) [file pone.0177751.s004.pdf]

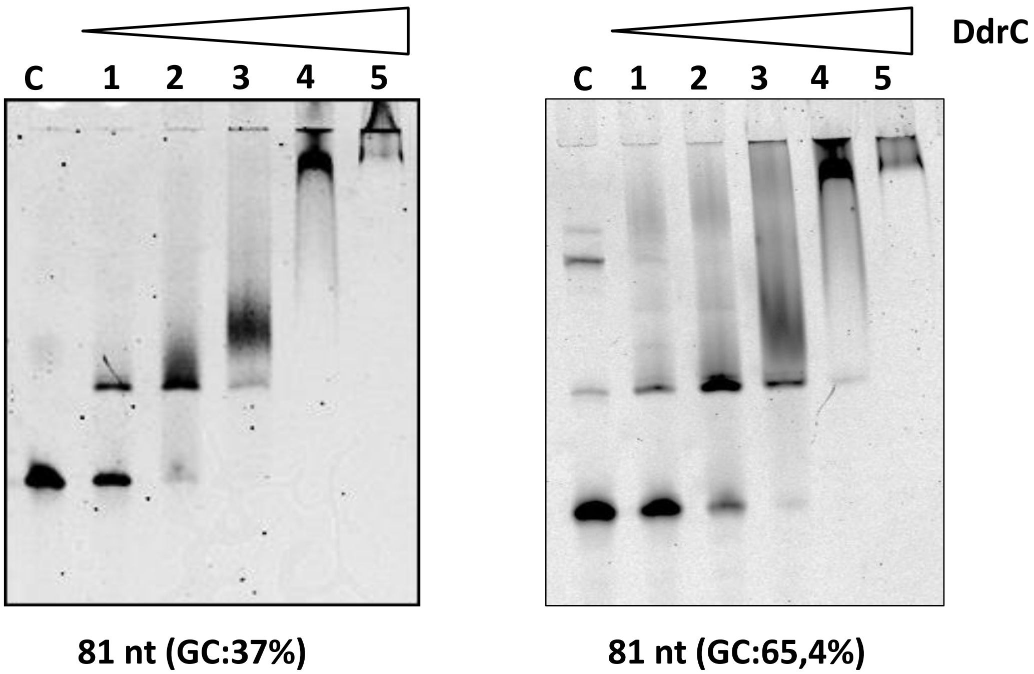

S4 Fig. DdrC binds to ssDNA without preference of DNA sequence.

Two 81-mer fluorescent oligonucleotides containing 37% or 65.4% GC were incubated with increasing concentrations of DdrC (0.175 µM, 0.35 µM, 0.7 µM, 1.4 µM, 2.8 µM) (lanes 1-5). C: control DNA without protein. Reaction products were separated on a 6% native polyacrylamide gel.

Oligonucleotides sequence:

Cy5gattaccacgaggagtatcttaagaattctgatatacttgaagaagacatacaggaacaagaacaaagactgaacgagctt (37%GC)

Cy5gttgctggacgtacagcgccactatggccgcctgggctggaccagcggcgagattccgtccgggggctaccagtttcccat (65.4%GC).
