# Supplementary material for: In vivo and in vitro characterization of DdrC, a DNA damage response protein in Deinococcus radiodurans bacterium
Source: PLoS One. 2017 May 18;12(5):e0177751. doi: 10.1371/journal.pone.0177751 (PMC5436757; doi:10.1371/journal.pone.0177751)
Supplement: S5 Fig — (PDF) [file pone.0177751.s005.pdf]

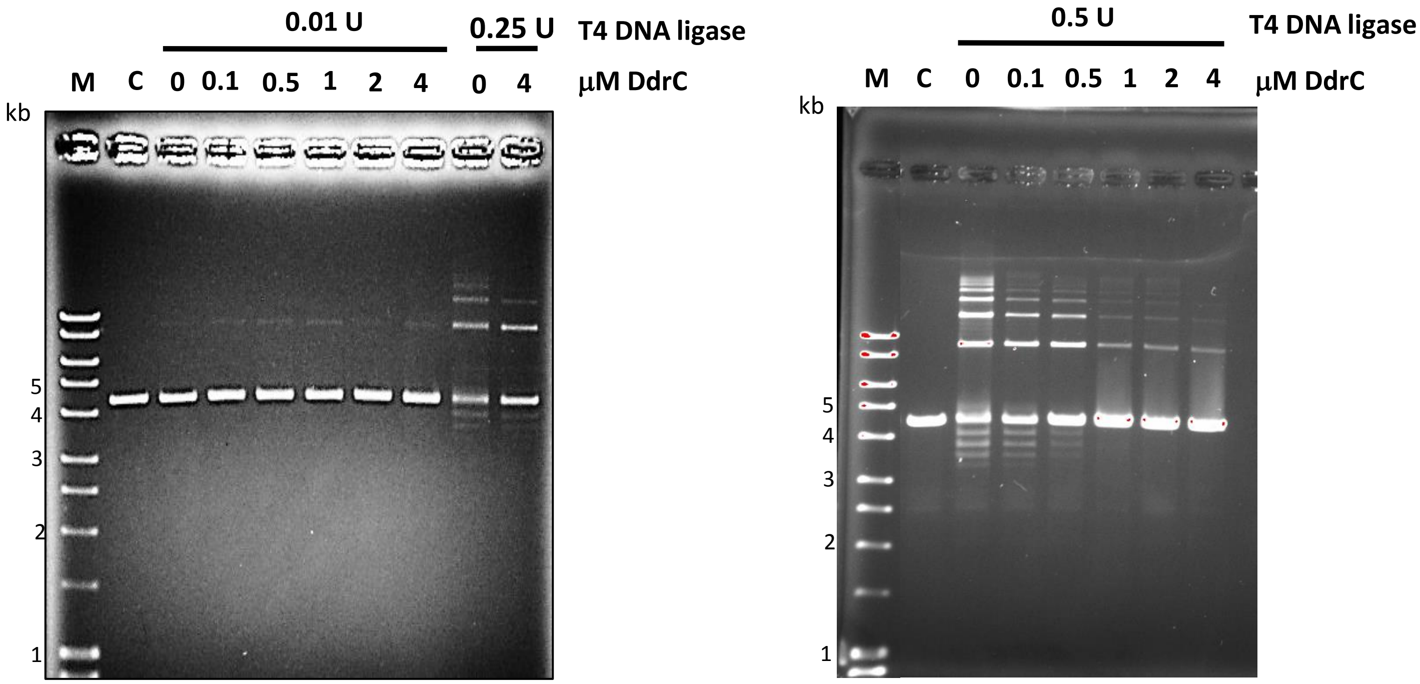

S5 Fig. DdrC protein does not stimulate DNA ligation of cohesive ends by T4 DNA ligase

Linear dsDNA (PstI-digested pBR322, 200 ng) was preincubated in the absence or the presence of the indicated DdrC protein concentrations for 15 min at 4°C prior addition of T4 DNA ligase. The ligation assays were performed with 3 different concentrations (0.01 U, 0.25 U, 0.5 U) of T4 DNA ligase at 30°C for 15 min. The reactions were stopped by addition of proteinase K and SDS and samples were subjected to electrophoresis through a 1% agarose gel.
