# Supplementary material for: In vivo and in vitro characterization of DdrC, a DNA damage response protein in Deinococcus radiodurans bacterium
Source: PLoS One. 2017 May 18;12(5):e0177751. doi: 10.1371/journal.pone.0177751 (PMC5436757; doi:10.1371/journal.pone.0177751)
Supplement: S1 Table — (PDF) [file pone.0177751.s006.pdf]

Bacterial strains	Description	Source or reference
D. radiodurans		
R1	Wild type, ATCC 13939	Laboratory stock
GY 15929	*ΔddrCΩkan*	This work
GY 15921	*ddrC::HA::kan*	This work
GY 15931	*ddrC::GFP::cat*	This work
GY 15928	*ddrC::Cherry ::kan*	This work
GY 15950	*ΔirrEΩcat*	This work
GY 15967	*ddrC::HA::kan ΔirrEΩcat*	This work
GY 15971	*ΔuvrAΩcat*	This work
GY 15972	*ΔuvsEΩhygro*	This work
GY 15973	*ΔddrCΩkan ΔuvrAΩcat*	This work
GY 15974	*ΔddrCΩkan ΔuvsEΩhygro*	This work
GY 15977	*ΔuvrAΩcat ΔuvsEΩhygro*	This work
GY 15978	*ΔddrCΩkan ΔuvrAΩcat ΔuvsEΩhygro*	This work
GY 16901	*ddrC::6His::cat*	This work
GY16902	*ddrC ::6His::cat ΔuvsEΩhygro*	This work
GY16903	*ddrC::HA::kan ΔuvsEΩhygro*	This work
GY15180	*ΔrecAΩcat*	Laboratory stock
GY15965	*ΔrecAΩcat ΔddrCΩkan*	This work
GY14164	*ΔddrOΩcat/p11891(prepUTs::ddrO*$^{+}$*)*	[23]
GY16917	[*ddrC::HA::kan ΔddrOΩcat/p11891(prepUTs::ddrO*$^{+}$*)*]	This work
GY16905	*ddrC::Cherry ::kan ΔuvsEΩhygro*	This work
GY16906	*ddrC::GFP::cat ΔuvsEΩhygro*	This work

E. coli		
DH5α	*supE44 ΔlacU(Δ80lacZΔM15) hsdR17 recA1 endA1 gyrA96 thi1-relA1*	Laboratory stock
Rosetta2(DE3)pLysS	*F- ompT hsdSB(rB-mB-) gal dcm (DE3) pLysSRARE2 (CamR)*	Novagen
**Plasmids**		
p11086	Source of *kan* cassette	Laboratory stock
pPS6	Source of *cat* cassette	Laboratory stock
p12625	Source of *hygro* cassette	Laboratory stock
p12764	Source of *HA::kan* cassette	Laboratory stock
pFAP223	Source of *GFP::cat* cassette	Laboratory stock
pFAP208	Source of *Cherry*::*kan* cassette	Laboratory stock
p11891	p13841*:*prep*UTs::ddrO*	[23]
pET26b	pET expression system, *pT7lac*, C-terminal 6His-tag	Novagen
pET26b-*ddrC*	pET26b *NdeI/XhoI*+ PCR fragment containing *ddrC*	This work

S1 Table: Bacterial strains and plasmids
