# Supplementary material for: In vivo and in vitro characterization of DdrC, a DNA damage response protein in Deinococcus radiodurans bacterium
Source: PLoS One. 2017 May 18;12(5):e0177751. doi: 10.1371/journal.pone.0177751 (PMC5436757; doi:10.1371/journal.pone.0177751)
Supplement: S2 Table — (PDF) [file pone.0177751.s007.pdf]

**Primers**	**Sequence**
**Primers used for inactivation of *DdrC* (strain GY15929)**	
DdrC1	TTCGCAGGCCGACCAGATAC
DdrC2Bam	ATGGATCCCAGCGGAGCGTTCTTCATAC
DdrC7Xba	AATTCTAGATGCAGCGCGTAGGCTGAGAC
DdrC8	GCAGCAGTTCGAGATAATGG
**Primers used to construct *DdrC* ::*HA*::*kan* (strain GY15921), *ddrC*::*GFP*::*cat* (strain GY15931), *ddrC*::*Cherry*::*kan* (strain GY15928)**	
DdrC5	TGAGCGTGCCCGATTTCGTG
DdrC6Bam	ATGGATCCGCCTACGCGCTGCACCTGGC
DdrC7Xba	AATTCTAGATGCAGCGCGTAGGCTGAGAC
DdrC8	GCAGCAGTTCGAGATAATGG
**Primers used for inactivation of *irrE* (strain GY15950)**	
IrrE1	CGTGCTCCTCAAGCTCTACG
IrrE2Bam	GCATGGATCCCAGTGATTTCGCCTGTTTTG
IrrE3Xba	GCATTCTAGAGTGAACTGGACGGGCCGTAT
IrrE4	GGTTGTGGTCCAGCGTCTTG
**Primers used for inactivation of *uvrA1* (strain GY15971)**	
UvrA9	GCGCAAGCTGATGGCATGAC
UvrA10Bam	ATGCGGATCCGAAGCTCCTTTTGGCTGTCC
UvrA7Xba	GGAATCTAGAATAGCGGTTGCGGTTGAAGC
UvrA8	CATAGATCAGGCCGGCGAAG
**Primers used for inactivation of *uvsE* (strain GY15972)**	
UvsE1	AAGCGACCCTCAGCACCTTC
UvsE2Bam	ATGCGGATCCTTCTTACGCCAAGAAAAGGG
UvsE3Xba	ATGTCTAGACGCCACTCGCACCTGATTGC
UvsE4	CATTCGACTCGCGCAAGATG
**Primers used to construct *ddrC::6his* at the locus (strain GY16901)**	
DdrC5	TGAGCGTGCCCGATTTCGTG
CB106bam	AGTGGATCCTCAGTGGTGGTGGTGGTGGTGCTCGAG
CB105Xba	AATTCTAGAGACAAGCGCCATAAAGAAGCTGCCC
DdrC4	TCAGCGTCTTGCGGTTGTCG
**Primers used to amplify *cat, kan, hygro* cassettes**	
PS177	ATTGGATCCTATACGGAACCTATACGGG
PS178	ATTTCTAGACGCGGCCGCACTTATTCA
Kan5	GGAAGGATCCGCATTCTGCTCCAGCATCTC
Kan3	GGAATCTAGAGCAAGCAGCAGATTACGCGCA
E87	AATTCTAGAGATCCGTGTTTCAGTTAGCC

E88	ATTGGATCCCGACGGCCAGTGAATTCGAG
**Primers used to amplify *HA::kan, GFP::cat, and Cherry::kan* cassettes**	
PS455	GACCGGATCCTACCCGTACGACGTGCCCGA
PS457	AAATTCTAGACGAATTGGGCCCGGTCTGAC
pCR21m	CACAGGAAACAGCTATGACC
pCR21v	AAACGACGGCCAGTGAATTG
**Primers used to amplify the *ddrC* gene for cloning in the pET26b expression vector**	
DdrC-Nde	GAGGCACAACATATGAAGAACGCTCCGCTGACCCT
DdrC-Xho	ACGTCTCGAGGCCTACGCGCTGCACCTGGC

S2 Table. Primers used in this study
